# Thymoma Type B2 Associated with Myasthenia Gravis Presenting as Progressive Respiratory Distress: A Case Report

**DOI:** 10.7759/cureus.110699

**Published:** 2026-06-11

**Authors:** Harun Iskandar, Dicky Rahmat, Nur Ahmad Tabri, Dewi Setiawati

**Affiliations:** 1 Internal Medicine, Hasanuddin University, Makassar, IDN; 2 Pulmonary Medicine, Hasanuddin University, Makassar, IDN; 3 Biomedical Sciences, Universitas Islam Negeri Alauddin Makassar, Makassar, IDN

**Keywords:** anterior mediastinal tumor, myasthenia gravis, paraneoplastic syndrome, thymectomy, thymoma

## Abstract

The association between thymoma and myasthenia gravis (MG) is well established; therefore, the significance of this case does not rest on the coexistence of these conditions alone. Its educational value lies in the diagnostic pathway, in which fluctuating myasthenic symptoms preceded tumor recognition, the initial chest radiograph did not demonstrate an evident mediastinal mass, and contrast-enhanced computed tomography (CECT) subsequently identified a necrotic anterior mediastinal lesion. A 54-year-old woman presented with progressive dyspnea, generalized fatigable weakness, dysphagia, facial muscle cramps, and bilateral eyelid heaviness. Her symptoms worsened with physical activity and improved with rest and pyridostigmine therapy, supporting a clinical diagnosis of MG. Preoperative acetylcholine receptor antibody testing, muscle-specific kinase antibody testing, repetitive nerve stimulation, and baseline electromyography were not documented in the available case records. Chest radiography demonstrated cardiomegaly with aortic dilatation and elongation, but no definite mediastinal mass. Thoracic CECT revealed a well-circumscribed, non-calcified anterior mediastinal isodense lesion with post-contrast enhancement and central necrosis, measuring approximately 3.34 × 2.03 × 2.99 cm. Bronchoscopy was performed as an adjunctive respiratory workup and showed hyperemic and cicatricial mucosa with mucopurulent secretions; cytology showed no malignant cells, and aerobic culture showed no growth. The patient underwent wide excision of a left superior anterior mediastinal mass through an anterolateral thoracotomy approach. Histopathology confirmed WHO type B2 thymoma with invasion into connective and adipose tissue, most consistent with Masaoka-Koga stage II. The patient had favorable early postoperative neurological improvement, and postoperative electromyography showed no persistent neuromuscular transmission abnormality. This case suggests that clinically typical MG may provide an early diagnostic clue to occult thymoma even when chest radiography is unrevealing. Because this is a single case report and long-term follow-up data were not available in the source documentation, the durability of neurological remission and oncological control should be interpreted cautiously and assessed through continued surveillance.

## Introduction

Thymoma is a rare thymic epithelial tumor and remains one of the most important neoplasms of the anterior mediastinum. Its clinical relevance extends beyond local mediastinal disease because thymoma may be accompanied by autoimmune and paraneoplastic manifestations. The WHO classification provides the histological framework for thymic epithelial tumors, while clinical staging remains essential for prognostic interpretation [1,2].

Among thymoma-associated immune manifestations, myasthenia gravis (MG) is the most clinically recognized. Approximately 10-20% of adult patients with MG have an underlying thymoma, whereas 30-50% of patients with thymoma develop MG. The pathogenesis is linked to impaired thymic immune tolerance, abnormal thymic epithelial cell function, and autoreactive T-cell mechanisms that promote antibody-mediated neuromuscular junction dysfunction [3,4].

Imaging is central to the evaluation of suspected anterior mediastinal lesions. Chest radiography may be normal or non-specific when lesions are small or anatomically subtle. Contrast-enhanced computed tomography (CECT) provides a more precise assessment of tumor size, enhancement pattern, necrosis, local invasion, and relationship to adjacent mediastinal structures [5,6].

Complete surgical excision remains the cornerstone of treatment for resectable thymoma, but interpretation of outcome requires attention to surgical approach, completeness of resection, histological subtype, staging, perioperative course, and follow-up duration [7,8]. We report a case of WHO type B2 thymoma associated with clinically diagnosed MG in which myasthenic symptoms preceded radiological identification of an occult anterior mediastinal mass. The purpose of this report is to highlight the diagnostic value of cross-sectional imaging and to present a balanced interpretation of early postoperative neurological improvement.

## Case presentation

A 54-year-old woman was referred to Dr. Wahidin Sudirohusodo Hospital, Makassar, Indonesia, with progressive shortness of breath for one month, which had worsened markedly during the preceding week. Dyspnea occurred predominantly during physical activity and was not influenced by posture or weather changes. The patient also reported intermittent productive cough with whitish sputum, generalized weakness involving all extremities, dysphagia, facial muscle cramps, and bilateral eyelid heaviness.

The neuromuscular symptoms had been present for approximately three months and had progressively worsened over the preceding two months. Weakness became more pronounced after activity and improved with rest. The patient had previously been evaluated by a neurologist and was clinically diagnosed with MG based on fluctuating fatigable weakness, ptosis, bulbar symptoms, and symptomatic improvement after pyridostigmine therapy. She had been receiving pyridostigmine 60 mg three times daily for two months with partial symptomatic relief. Preoperative acetylcholine receptor antibody testing, muscle-specific kinase antibody testing, repetitive nerve stimulation, and baseline electromyography were not documented in the available case records. Therefore, the diagnosis of MG was based on the characteristic clinical phenotype, neurological assessment, and therapeutic response rather than serological or electrophysiological confirmation.

The patient denied fever, hemoptysis, chest pain, or previous tuberculosis exposure. She had a history of hypertension treated irregularly with amlodipine 5 mg daily. There was no family history of malignancy or autoimmune disease. Physical examination showed mild dyspnea, symmetrical chest expansion, normal tactile fremitus, sonorous percussion bilaterally, and vesicular breath sounds without wheezing or crackles.

Neurological examination demonstrated bilateral ptosis and fluctuating generalized weakness, which became more prominent after repeated muscle activity.

Laboratory evaluation showed leukocytosis of 12,200/µL with neutrophil predominance of 65.7%, while other blood chemistry parameters were within normal limits. The Xpert MTB/RIF assay, an automated cartridge-based nucleic acid amplification test used to detect *Mycobacterium tuberculosis* complex and rifampicin resistance, yielded a negative result. Bronchoscopic cytology from brushing and bronchial washing showed no malignant cells, and aerobic bacterial culture showed no growth.

Chest radiography performed on July 21, 2023, demonstrated cardiomegaly with dilatation and elongation of the aorta without an evident mediastinal mass (Figure 1). Thoracic CECT performed on July 25, 2023, revealed a well-circumscribed, regular-edged, non-calcified isodense lesion in the anterior mediastinum.

**Figure 1:**
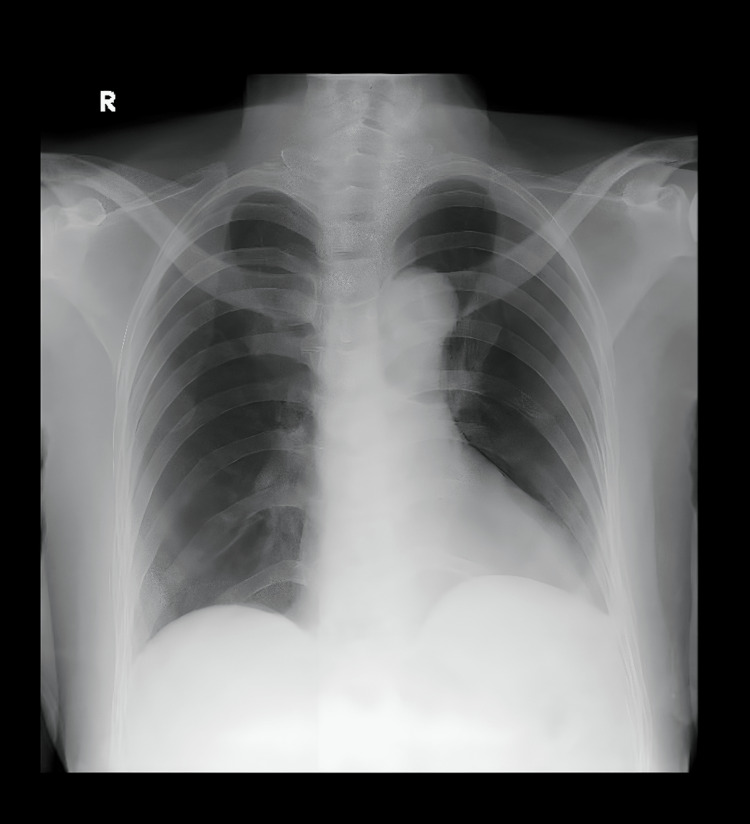
Chest radiograph showing cardiomegaly with dilatation and elongation of the aorta. No evident mediastinal mass is seen on initial radiography.

The lesion enhanced after contrast administration, measured approximately 3.34 x 2.03 x 2.99 cm, and contained central necrosis (Figure 2). Additional CECT findings included pulmonary consolidation with air bronchogram and fibrotic lines in both lower lung regions.

**Figure 2:**
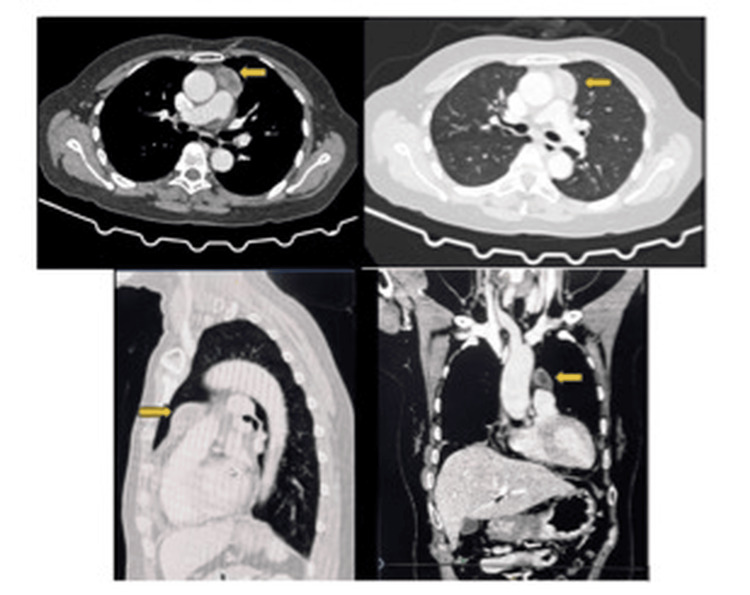
Contrast-enhanced chest computed tomography showing a well-circumscribed enhancing anterior mediastinal lesion with central necrosis, suggestive of thymoma.

Bronchoscopy was performed on July 28, 2023, because the patient had productive cough, pulmonary consolidation, and mucopurulent airway secretions, requiring evaluation for airway infection, tuberculosis, and endobronchial malignancy. The examination demonstrated hyperemic and cicatricial mucosa in the left upper lobe, and brushing was performed in segment B1 of the left upper lobe (Figure 3). Although bronchoscopy did not directly establish the diagnosis of the anterior mediastinal mass, it contributed to excluding relevant airway and infectious differential diagnoses.

**Figure 3:**
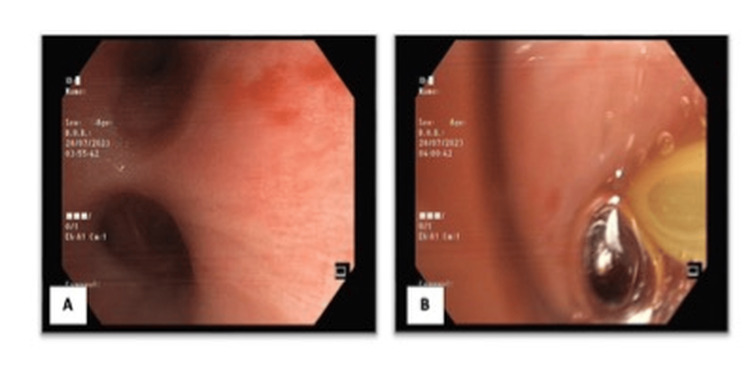
Bronchoscopic findings showing hyperemic and cicatricial mucosa of the left upper lobe; brushing was performed in segment B1 to evaluate respiratory differential diagnoses.

Based on fluctuating myasthenic symptoms, partial response to pyridostigmine, and CECT evidence of an anterior mediastinal lesion, the patient was diagnosed with suspected thymoma-associated MG. Surgery was performed on August 10, 2023, for diagnostic and therapeutic purposes. The patient underwent wide excision of the left superior anterior mediastinal mass through an anterolateral thoracotomy approach. Intraoperatively, the mass measured approximately 6 × 6 cm and was removed for histopathological examination (Figure 4). The discrepancy between the CECT measurement and the intraoperative gross measurement was interpreted as reflecting different measurement contexts: the radiological measurement represented the enhancing lesion visible on cross-sectional imaging, whereas the operative measurement likely reflected the gross resected mass together with the capsule, thymic tissue, perithymic fibroadipose tissue, and tissue-handling effects.

**Figure 4:**
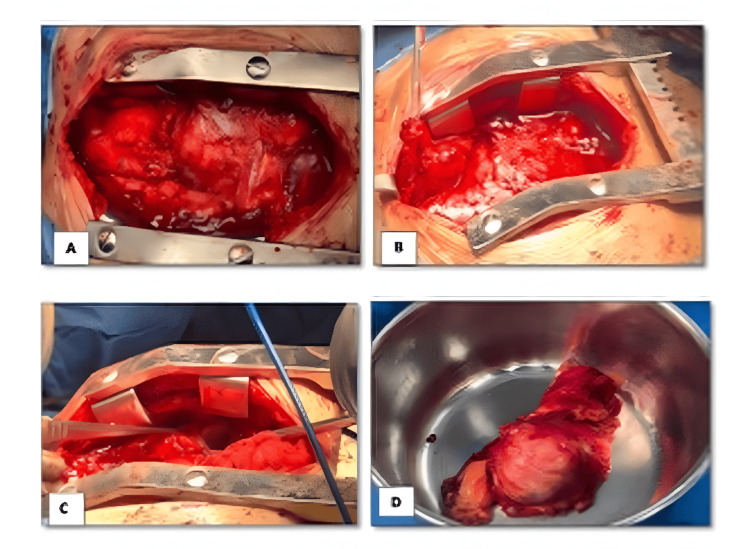
Intraoperative findings during left anterolateral thoracotomy and wide excision of the left superior anterior mediastinal mass. (A) Exposure of the anterior mediastinal mass. (B) Tumor mobilization and removal. (C) Wide excision of the tumor bed. (D) Gross appearance of the excised mediastinal mass. Orientation markers indicating cranial, caudal, right, and left directions have been added to improve anatomical clarity.

Histopathological examination reported on August 21, 2023, confirmed WHO type B2 thymoma. Microscopically, the tumor was composed predominantly of lymphocytes, with clustered large polygonal epithelial cells showing round to vesicular nuclei. Numerous perivascular spaces were present. At the tumor edge, connective tissue was identified, and several foci showed invasion into connective and adipose tissue (Figure 5). Based on invasion into surrounding connective and adipose tissue without documented invasion into adjacent organs or pleural/pericardial dissemination, the tumor was classified as Masaoka-Koga stage II according to the International Thymic Malignancy Interest Group-adopted Masaoka-Koga staging system. Because the available case documentation did not include sufficient information to assign a complete TNM category, Masaoka-Koga staging was used for clinicopathological interpretation. Immunohistochemical staining was not available in the source documentation; therefore, the diagnosis was based on the reported morphology on hematoxylin and eosin staining and the final pathology conclusion. Microscopic margin status was not explicitly stated, so the resection could not be definitively classified as R0 or R1.

**Figure 5:**
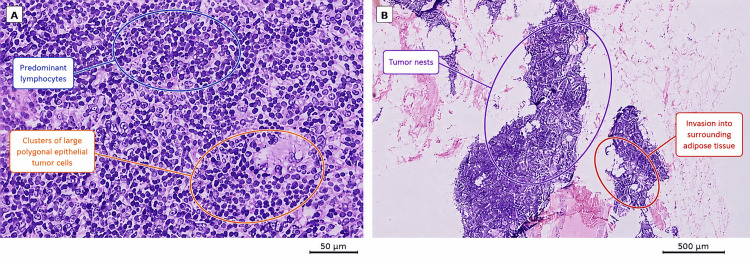
Histopathological examination of WHO type B2 thymoma. (A) Predominant lymphocytes with clusters of large polygonal epithelial tumor cells. (B) Tumor nests with invasion into surrounding adipose tissue. Hematoxylin and eosin staining; scale bars: A = 50 µm and B = 500 µm.

Postoperatively, the patient was monitored for respiratory deterioration and myasthenic worsening. No postoperative myasthenic crisis or respiratory failure was documented in the available records. Ptosis, generalized weakness, and dysphagia resolved clinically after surgery. Follow-up electromyography after thymectomy demonstrated no persistent neuromuscular transmission abnormality. Because preoperative electrophysiological data were not available, the postoperative electromyography result was interpreted as supportive evidence of early neurological recovery rather than a direct preoperative-to-postoperative comparison. The clinical timeline is summarized in Table 1.

**Table 1:** Clinical, diagnostic, and therapeutic timeline.

Date	Event	Key findings or relevance
July 21, 2023	Chest radiography	Cardiomegaly with dilatation and elongation of the aorta; no evident mediastinal mass.
July 25, 2023	Thoracic contrast-enhanced computed tomography (CECT)	Well-circumscribed, enhancing anterior mediastinal lesion with central necrosis measuring approximately 3.34 × 2.03 × 2.99 cm.
July 28, 2023	Bronchoscopy	Hyperemic and cicatricial mucosa in the left upper lobe; bronchial brushing and washing showed no malignant cells; aerobic culture showed no growth.
August 10, 2023	Surgery	Left anterolateral thoracotomy with wide excision of a 6 × 6 cm mass in the left superior anterior mediastinum.
August 21, 2023	Histopathology	WHO type B2 thymoma with lymphocyte-rich morphology, polygonal epithelial cells, perivascular spaces, and focal invasion into connective and adipose tissue.
Early postoperative period	Clinical and electrophysiological outcome	Resolution of ptosis, generalized weakness, and dysphagia; postoperative electromyography showed no persistent neuromuscular transmission abnormality.

## Discussion

This case illustrates an established but clinically important association between thymoma and MG, while its educational value lies in the diagnostic sequence. The patient had a typical fluctuating myasthenic phenotype, including fatigable weakness, ptosis, and dysphagia, but the initial chest radiograph did not reveal a mediastinal mass. The anterior mediastinal lesion was identified only after CECT, supporting the role of cross-sectional imaging when clinical suspicion remains high despite unrevealing radiography.

Thymoma-associated MG is part of a broader spectrum of thymoma-related immune dysregulation. Paraneoplastic syndromes in thymoma may include MG, pure red cell aplasia, hypogammaglobulinemia or Good syndrome, nephrotic syndrome, Morvan syndrome, and other immune-mediated manifestations [9]. In the present patient, MG was the dominant paraneoplastic neurological manifestation. This is clinically relevant because paraneoplastic neurological syndromes may precede tumor detection and can serve as early warning signs of an occult malignancy.

The absence of documented preoperative acetylcholine receptor antibody testing, muscle-specific kinase antibody testing, repetitive nerve stimulation, and baseline electromyography is a limitation. Nevertheless, the diagnosis of MG was clinically coherent because the patient had fatigable weakness, ptosis, bulbar involvement, improvement with rest, and response to pyridostigmine. In settings where full serological and electrophysiological testing is not immediately available, this pattern remains an important basis for clinical suspicion, although it should be acknowledged as less definitive than antibody- or electrophysiology-confirmed MG.

The diagnostic contribution of bronchoscopy should also be interpreted correctly. Bronchoscopy was not intended to diagnose the mediastinal mass directly. It was justified by the patient’s respiratory symptoms, pulmonary consolidation, and mucopurulent secretions, and it helped exclude endobronchial malignancy, tuberculosis-related airway disease, and bacterial infection. The negative cytology and culture results supported the decision to focus further diagnostic interpretation on the anterior mediastinal lesion identified by CECT.

Compared with previously reported cases of thymoma-associated MG, the present case shares typical features such as adult onset, fluctuating neuromuscular symptoms, anterior mediastinal tumor detection, histopathological confirmation, surgical management, and early postoperative improvement. Kumaravel and Sonti described thymoma with myasthenic symptoms in cases where mediastinal tumors were radiologically evident; Abrar et al. reported a more severe presentation involving myasthenic crisis and intensive post-crisis care; Huang et al. described thymoma-associated MG with autoimmune overlap and inflammatory myopathy [10-12]. In contrast, the present case is distinguished by the absence of an evident mediastinal mass on initial radiography, subsequent CT detection of a necrotic anterior mediastinal lesion, and early clinical recovery after tumor excision.

Histopathology is central to diagnosis and prognostic interpretation. The reported features, including abundant lymphocytes, clustered large polygonal epithelial cells, perivascular spaces, and invasion into connective and adipose tissue, support WHO type B2 thymoma. Immunohistochemical staining would have provided additional confirmation and differential diagnostic support, particularly through epithelial markers and lymphoid immaturity markers. However, the available pathology report provided a clear morphological diagnosis of type B2 thymoma, and the absence of immunohistochemistry was therefore treated as a limitation rather than a reason to reject the pathological diagnosis.

Staging and completeness of resection are critical for prognosis. The presence of invasion into surrounding adipose tissue is most consistent with Masaoka-Koga stage II when there is no documented invasion into adjacent organs or pleural/pericardial dissemination. The operative record described wide excision and removal of the mass, but microscopic margin status was not available; therefore, the final manuscript should not claim confirmed R0 resection unless margin negativity is documented. This cautious wording preserves scientific accuracy and avoids overstatement.

Although the eighth-edition TNM system is recommended for standardized staging of thymic epithelial tumors, the International Thymic Malignancy Interest Group has also adopted the Masaoka-Koga system as a clinically useful and widely used staging framework. In the present case, the available pathological information documented invasion into surrounding connective and adipose tissue, without documented invasion into adjacent organs or pleural/pericardial dissemination. These findings were most consistent with Masaoka-Koga stage II disease. A complete TNM stage could not be assigned because nodal assessment and complete metastatic staging details were not available in the source documentation.

Continued surveillance was defined as structured long-term clinical, neurological, and radiological follow-up after tumor resection. For this patient, continued surveillance should include neurological assessment for recurrent myasthenic symptoms and thoracic imaging to monitor for thymoma recurrence. In line with published follow-up recommendations for resected thymoma, a reasonable surveillance plan would include baseline postoperative chest computed tomography within three to four months after surgery, followed by annual chest computed tomography for five years and then every two years for a total follow-up duration of 10-15 years, particularly if complete resection is confirmed. If the final resection status is uncertain or if recurrence risk is considered higher, closer imaging surveillance may be justified.

The surgical approach was specified to clarify the operative strategy. Although median sternotomy is commonly used for thymoma because it provides broad exposure of the anterior mediastinum, the optimal approach should be individualized according to tumor location, size, suspected invasion, adhesions, and the need for safe intraoperative control. In this case, an anterolateral thoracotomy approach was used to access the left superior anterior mediastinal mass and allowed wide excision under direct visualization. This approach is clinically relevant because it can provide flexible exposure and may be extended to wider thoracic approaches when adhesions or involvement of adjacent mediastinal structures are encountered.

Type B2 thymoma has more relevant prognostic implications than lower-risk histological subtypes, particularly when invasion beyond the capsule or into perithymic tissue is present. For this reason, early postoperative symptom resolution should not be equated with definitive long-term remission. Postoperative surveillance should address both oncological recurrence and neurological status. Guideline-based follow-up typically requires serial clinical evaluation and thoracic imaging, while MG outcomes are best assessed using standardized neurological status scales when available [13].

The neurological outcome in this patient was favorable in the early postoperative period, with resolution of ptosis, generalized weakness, and dysphagia. Postoperative electromyography showed no persistent neuromuscular transmission abnormality. However, because preoperative electrophysiological data and long-term follow-up were not documented, the result should be interpreted as early postoperative improvement rather than durable remission. This distinction directly addresses the limitations inherent to a single case report and keeps the conclusion appropriately balanced.

## Conclusions

In adult patients with MG, thymoma should remain an important diagnostic consideration, and thymoma may present with MG as a paraneoplastic neurological manifestation. This case suggests that fluctuating myasthenic symptoms may provide an early clinical clue to occult anterior mediastinal thymoma, even when initial chest radiography is unrevealing. Early CECT, careful exclusion of respiratory differential diagnoses, histopathological confirmation, surgical excision, staging, and multidisciplinary management may contribute to favorable early outcomes. Because the report describes a single patient and lacks documented preoperative electrophysiology, immunohistochemistry, definitive microscopic margin status, and long-term follow-up, the durability of neurological remission and oncological control should be interpreted cautiously. Continued surveillance should include structured neurological follow-up and serial thoracic imaging, with a suggested duration of 10-15 years to monitor for late thymoma recurrence and recurrent myasthenic symptoms.
